# DMI-Fungicide Resistance in *Venturia nashicola*, the Causal Agent of Asian Pear Scab—How Reliable Are Mycelial Growth Tests in Culture?

**DOI:** 10.3390/microorganisms9071377

**Published:** 2021-06-24

**Authors:** Hideo Ishii, Hans Jorgen Cools, Kumiko Nishimura, Lorenzo Borghi, Kenji Kikuhara, Yuichi Yamaoka

**Affiliations:** 1 Faculty of Life and Environmental Sciences, University of Tsukuba, Tennodai 1-1-1, Tsukuba 305-8572, Japan; yamaoka.yuichi.gp@u.tsukuba.ac.jp; 2 National Institute for Agro-Environmental Sciences, Kannondai 3-1-3, Tsukuba 305-8604, Japan; artanaria5@yahoo.co.jp; 3 Department of Agriculture, Kibi International University, Sareo 370-1, Shichi, Minami-Awaji 656-0484, Japan; 4 Syngenta, Jealott’s Hill International Research Centre, Bracknell RG42 6EY, UK; hans.cools@syngenta.com; 5 Syngenta Crop Protection AG, Werk Stein, Schaffhauserstrasse, WST.820.2.79, CH-4332 Stein, Switzerland; Lorenzo.Borghi@syngenta.com; 6 Fukuoka Agriculture and Forestry Research Center, Yoshiki 587, Chikushino 818-8549, Japan; kikuhara@farc.pref.fukuoka.jp

**Keywords:** CYP51, DMIs, fungicide resistance, pear scab, sensitivity tests, *Venturia nashicola*

## Abstract

Scab, caused by *Venturia nashicola*, is among the most serious diseases of Asian pears and control of this disease largely relies on sterol demethylation inhibitor (DMI) fungicides. However, pear growers have complained about field performance of DMIs since the mid-2000s. In this study, to evaluate pathogen sensitivity, mycelial growth tests and inoculation tests were conducted using DMI-amended culture medium and fungicide-sprayed potted pear trees, respectively. Results confirmed distribution of isolates resistant to fenarimol, hexaconazole, and difenoconazole in the field populations. Importantly, results from tests in culture did not fully correlate with those from tests in planta. Due to phenotypic instability of resistance and poor sporulation of this pathogen in culture, resistance is generally assessed by laborious and time-consuming inoculation with conidia collected from a field. To improve the result interpretation from in vitro tests, the isolates were genotyped: the *CYP51* gene which encodes the target sterol 14α-demethylase was sequenced and various mutations have been detected in the coding sequence of DMI-resistant isolates. In addition to the detected single nucleotide polymorphisms, alternative mechanisms, not based on changes in the structure of the target protein, may also increase DMI resistance. Development of molecular methods for the diagnosis of DMI resistance seems to be challenging in *V*. *nashicola*.

## 1. Introduction

Asian pears such as Japanese pear (*Pyrus pyrifolia* var. *culta*) and Chinese pears (*P. bretschneideri* and *P. ussuriensis*), distinct from European pear (*P. communis*), are widely grown in East Asia [1]. Scab, caused by the ascomycete *Venturia nashicola* [2,3], is among the most serious diseases of Asian pears [4] but is not known to occur outside Asia. Consequently, *V. nashicola* is a quarantined pathogen in many countries [5,6].

To control this disease, growers largely rely on spray applications of fungicides because there are very few commercially acceptable cultivars with scab resistance [4,7,8]. However, the frequent use of benzimidazole (MBC) fungicides resulted in resistance development in the pathogen in the mid-1970s [9,10]. As a result, various sterol demethylation inhibitor (DMI) fungicides including triflumizole, bitertanol, fenarimol, hexaconazole, fenbuconazole, difenoconazole and others, targeting the P450 sterol 14α-demethylase (CYP51) protein [11,12], have been registered and sprayed during blossom and after flowers fall since 1986 in Japan [10,13].

Sterols such as ergosterol, ergosta-5,24(24^1^)-dien-3β-ol, and ergosta-5,7-dien-3β-ol are important constituents of fungal cell membranes regulating their stability and permeability [14,15,16]. Therefore, fungicides inhibiting key enzymes involved in fungal sterol biosynthesis including CYP51 have been very effective [11] with DMIs sharing over 30% of the fungicide market in agriculture worldwide [12]. Resistance to DMIs evolves slowly and resistance levels are often low compared with those for MBC resistance [17]. Despite that, DMI resistance was reported in many pathogens including the related fungus *V. inaequalis*, which causes apple scab, in as early as the 1980s [18,19,20]. In Japan, to maintain their field performance, it has been advised to limit DMI applications to a maximum of three times per year in a mixture or alternative use with other effective fungicides having a different mode action [13,21]. As a consequence, no clear evidence of DMI resistance had been shown for pear scab until the mid-2000s, about 20 years after their introduction to Japan [10,22].

Meanwhile, methods for testing *V*. *nashicola* sensitivity to two DMIs triflumizole and bitertanol were developed. A mycelial growth test on fungicide-amended culture medium and comparison of EC_50_ (50% effective concentration to inhibit growth) rather than MIC (minimum inhibitory concentration) values was recommended [23]. Using this method, baseline sensitivity was also determined for fenarimol and less-sensitive isolates were detected occasionally in culture, although the performance of DMIs was maintained in planta [24,25]. In the spring 2005, however, pear trees were heavily attacked by scab in commercial orchards located across Fukuoka Prefecture, the southwest of Japan, where growers tended to apply DMIs more frequently than recommended in a spray calendar [26]. Therefore, we tested DMI sensitivity of *V*. *nashicola* isolates and continued to monitor change of sensitivity in field populations.

Various mechanisms are known to be involved in DMI resistance. They include [11,12] (1) modifications in target enzyme caused by mutations of the *CYP51* genes resulting in decreased binding affinity of fungicides, (2) overexpression of *CYP51* genes, and (3) decreased intracellular fungicide concentration mediated by increased energy-dependent efflux. For *V*. *nashicola*, Cools et al. [25] cloned and sequenced the complete *CYP51* gene but no alterations were found in the isolates with reduced DMI sensitivity compared with those of wild-type sensitive isolates. Resistance monitoring is extremely laborious and time-consuming for *V. nashicola* isolates because their growth is slow both in culture and in planta. However, molecular mechanism(s) of DMI resistance are currently unknown for this fungus, making it difficult to develop DNA-based methods for diagnosis of resistance.

Therefore, the major objectives of this study were to (1) demonstrate and monitor DMI resistance of *V*. *nashicola* both in planta and in culture, (2) examine field relevance of reduced sensitivity in culture, (3) confirm instability of resistance, and (4) analyze sequences of the *CYP51* gene of resistant and sensitive isolates.

## 2. Materials and Methods

### 2.1. Fungal Isolation and Fungicides

Fifty-six single-spore stock isolates of *V*. *nashicola* originating from Hebei, China, were used to establish the baseline sensitivity to difenoconazole and hexaconazole. They were originally isolated in 1993 from an orchard where DMI fungicides had never been sprayed and tested for baseline fenarimol sensitivity [24]. Imported samples of leaves were handled according to the Plant Protection Act under the supervision of the Ministry of Agriculture, Forestry and Fisheries (MAFF), Japan. Next, pear leaves or fruit naturally infected with scab were collected arbitrarily from DMI-exposed commercial orchards located in Fukuoka and Saga prefectures, Japan, between 2005 and 2018, and 41 to 52 single-spore isolates obtained from each orchard. Conidia scraped directly from the lesions were suspended in sterile distilled water (DW) using a steel needle. Drops of conidial suspensions were placed on acidified 3% water agar (Wako, Osaka, Japan) plates and incubated at 15 °C for 5 to 7 days in the dark. Agar blocks containing single germinated conidia were individually cut with a steel needle after microscopic observation and transferred onto potato dextrose agar (PDA, Difco Laboratories, Franklin Lakes, NJ, USA) slants or plates amended with 50 mg/L of penicillin G potassium salt, 50 mg/L of streptomycin sulphate, and 0.05% (*v/v*) lactic acid. After incubation at 15 °C for approximately 2 months in the dark, pure cultures were established and stored at 4 °C until use. Formulated fungicides used in tests were fenarimol (Rubigan^®^ 12% wettable powder, Nissan Chemical, Tokyo, Japan), difenoconazole (Score^®^ 10% wettable powder, Syngenta), and hexaconazole (Anvil^®^ 2% flowable, Syngenta). These fungicides were all purchased.

### 2.2. Mycelial Growth Tests on Culture Medium

Single-spore isolates were transferred to PDA plates and incubated at 20 °C for about 45 days in darkness. Mycelial discs, 4 mm in diameter, were cut from an actively growing colony margin and transferred onto PDA plates containing each DMI fungicide at 0, 0.01, 0.05, 0.1, 0.5, 1, 5, 10, 50, and 100 mg/L of active ingredient (AI). After incubation at 20 °C in the dark for 21 days, the colony diameter (minus 4 mm for the disc) of two replicates per treatment was measured using a handheld digital caliper. The values of EC_50_ were calculated for each isolate by regressing percentage mycelial growth inhibition against the log of fungicide concentration using software supplied by K. So, ZEN-NOH (Tokyo, Japan). Two baseline-sensitive isolates Baoding 1-1 and Baoding 2-2, derived from Hebei, China, as described above, were included in the tests as a reference. The 95% confidence interval (95% CI) of average values, calculated by Microsoft Office Excel (Seattle, WA, USA), was compared. Statistical differences were found when the 95% CI of the average values did not overlap. The isolates were defined to be less-sensitive to fungicides when the EC_50_ values exceeded baseline sensitivity levels.

### 2.3. Conidia Formation in Culture

As conidia formation of *V. nashicola* is generally very poor on ordinary agar medium, conidia of this fungus were obtained in culture using the method of Parker et al. [27] after slight modification. This was the best method so far tested. Individual isolates were cultured on PDA plates at 20 °C in the dark for approximately 1 month. Mycelia were taken from the colony margins and, after removal of the agar medium, homogenized aseptically in sterile DW. Homogenates were spread over the surface of cellophane membranes, previously autoclaved and placed on PDA plates, and incubated at 20 °C for 1 month under black light blue irradiation.

### 2.4. Inoculation of Potted Pear Trees

For inoculum, conidia that formed on lesions of leaves or in culture were suspended in 0.01% (*v/v*) Tween 80 and 0.1% (*w/v*) sucrose in DW after washing with centrifugation [3]. As a DMI-sensitive reference, conidia were collected from trees at an experimental orchard in the National Institute for Agro-Environmental Sciences (NIAES), Tsukuba, Japan. The trees had no history of DMI spray treatments. Concentration of conidia was adjusted to approximately 2.5–5.0 × 10^5^ conidia mL^−1^ and 1.0 × 10^5^ mL^−1^ for those from lesions [3] and from culture [4], respectively. The suspensions were stored in a −30 °C or a −80 °C freezer until use. Young potted Japanese pear trees of Kousui (=Kosui), the most popular and widely grown cultivar in Japan, highly susceptible to scab, were treated using a hand-held sprayer until run-off with labelled concentrations of fungicides, fenarimol at 30 mg/L, hexaconazole at 10 mg/L, and difenoconazole at 25 mg/L of AI. DW was used as a control. One day after treatment, conidial suspensions were either sprayed on both sides of whole young leaves until run-off or dropped on to three sites for each main vein of five fresh leaves as a rule. Inoculated plants were incubated at 20 °C in a high humidity (RH: 95–100%) chamber (Koitotron™ TH; Koito Electric Industries, Shizuoka, Japan) for 48 h in the dark, and subsequently outside. One or two weeks after inoculation, the same fungicide and DW treatment was repeated because incubation period of scab fungus is 3 to 4 weeks long under optimal conditions and this might influence on residual fungicide efficacy. Three weeks and/or 1 month after inoculation, scab development was assessed visually based on the presence or absence of conidial formation on spray-inoculated leaves or droplet-inoculated sites and scab incidence (%) calculated as (number of sporulating leaves or sporulating sites/number of spray-inoculated leaves or droplet-inoculated sites) × 100. In case of spray inoculation, each leaf was assessed using the following scale: 0 = no visible symptoms; 1 = pinhole with no sporulation; 2 = chlorotic or necrotic lesions with no sporulation; 3 = moderately or sparsely sporulating lesions; and 4 = abundantly sporulating lesions, and disease severity was calculated as [(4*A* + 3*B* + 2*C* + *D*)/4E] × 100, where *A*, *B*, *C*, and *D* is the number of leaves corresponding to the scales, 4, 3, 2, and 1, respectively, and *E* is the total number of leaves assessed [28]. Control (%) by individual fungicides was calculated as [(incidence or disease severity on untreated trees − incidence or disease severity on treated trees)/incidence or disease severity on untreated trees] × 100. Resistance was defined based on reduction in fungicide efficacy in planta.

### 2.5. DNA Extraction, Polymerase Chain Reaction (PCR) Amplification and Sequence Analysis of the CYP51 Gene

Twenty-one single-spore isolates, obtained in 2005, 2017 and 2018, were selected based on the results from mycelial growth and inoculation tests performed in culture and in planta, respectively. Genomic DNA was extracted from culture of these isolates using the DNeasy Plant Mini Kit (Qiagen, Hilden, Germany) according to instructions from the manufacturer or the methods reported previously [29] after slight modification. In the latter case, a piece of PDA with actively growing mycelium was transferred in a 1.5 mL microtube containing 500 μL of lysis buffer [200 mM of Tris-HCl, 50 mM of ethylenediaminetetraacetic acid (EDTA), 200 mM of NaCl, and 1% *n*-lauroylsarcosine sodium salt, pH 8.0] and homogenized using a plastic pestle and an electric drill. The mixture was incubated at room temperature for 10 min and centrifuged at 13,000 rpm for 5 min at 4 °C. The supernatant (300 μL) was transferred to a fresh tube. After mixing with 750 μL of ethanol, the DNA was precipitated by centrifugation at 13,000 rpm for 2 min at 4 °C. The pellet was washed with 70% ethanol, air-dried in a laminar flow cabinet and dissolved in 50 μL of Tris-EDTA (TE) buffer containing 10 mM of Tris-HCl and 1 mM of EDTA (pH 8.0).

To amplify a partial fragment of the *CYP51* gene from genomic DNA, the polymerase chain reaction (PCR) primers specific for *V. nashicola*, Vn51seqF (5′-ATGGGACTCCTCTCTGCTCTCCTC-3′), Vn51seqF2 (5′-GCGCTTCTCATGGCCGGTCA-3′), and Vn51seqR (5′-CTATGATGATGACTTCTCTCTGCGT-3′) were designed by H. J. Cools in this study based on the nucleotide sequence of the National Center for Biotechnology Information (NCBI) GenBank accession: AJ314649 [25]. The 50 μL of PCR mixture contained 1 μL of genomic DNA, a set of forward (Vn51seqF or Vn51seqF2) and reverse (Vn51seqR) primers (0.2 μM for each), DNase-free water, and premixed Go Taq Green Master Mix (Promega, Madison, WI, USA). PCR was performed in a TaKaRa PCR thermal cycler (TaKaRa Bio, Kusatsu, Siga, Japan) or a Mastercycler nexus gradient (Eppendorf, Hamburg, Germany) programmed for 5 min at 94 °C, followed by 40 cycles of 1 min at 94 °C, 1.5 min at 50 °C or 60 °C, 2 min at 72 °C, a final extension for 4 min at 72 °C and holding at 4 °C. PCR products were separated by electrophoresis on a 1.5% agarose gel in TAE (40 mM Tris, 20 mM of acetic acid, and 1mM of EDTA) buffer and stained with GelRed^TM^ (Biotium, Hayward, CA, USA). PCR products, 1,688 bp (Vn51seqF and Vn51seqR) and 674 bp (Vn51seqF2 and Vn51seqR) in size, respectively, were cleaned using ExoSAP-IT (Affymetrix, Santa Clara, CA, USA) according to the instructions supplied by the manufacturer. Sequencing was conducted at Macrogen Japan (Kyoto, Japan) using the same primers as for PCR. After sequencing, the nucleotide and deduced amino-acid sequences were analysed with the NCBI/GenBank database (accession: AJ314649 and CAC85409, respectively) and using basic local alignment search tools (BLAST).

## 3. Results

### 3.1. Baseline Sensitivity to Difenoconazole and Hexaconazole in Culture

EC_50_ values of difenoconazole and hexaconazole were of 0.023 ± 0.142 mg/L and 0.007 ± 0.016 mg/L (average and 95% CI), respectively, when tested using 56 stock isolates originating from an orchard in China that had not received DMI applications. EC_50_ values of fenarimol, retested in this study, ranged from 0.050 mg/L to 1.038 mg/L with 0.142 ± 0.215 mg/L (average and 95% CI). These values represent baseline sensitivity. The average EC_50_ value of difenoconazole and hexaconazole was 0.017 mg/L and 0.009 mg/L, respectively, for baseline Korean isolates [30] and equivalent to the values shown in this study. No geographic variation was found in the baseline sensitivity between Chinese and Korean isolates.

### 3.2. Demonstration of Fenarimol Resistance in Planta and in Culture

**Figure 1:**
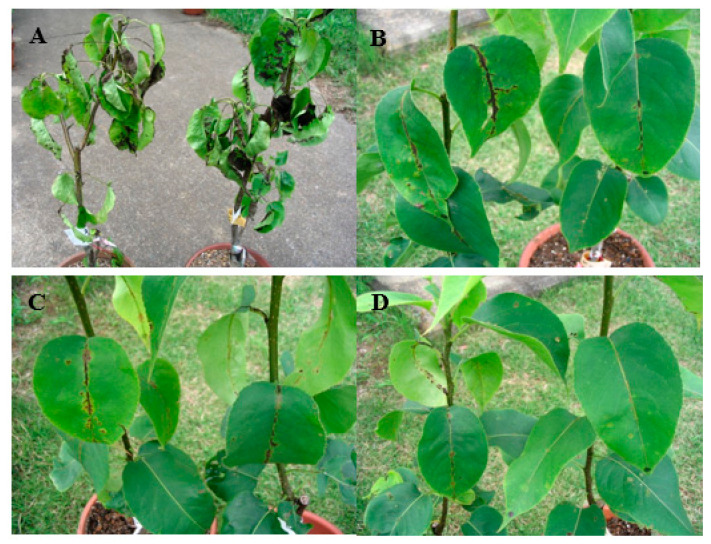
Control efficacy of fenarimol (on the right of each photo) sprayed preventatively and curatively at 30 mg/L AI against scab disease on potted Japanese pear trees (cultivar: Kousui). Distilled water was sprayed as a reference (on the left of each photo). Trees were inoculated with the conidial suspensions prepared from (**A**) naturally occurring sporulating lesions collected from Ukiha 2, (**B**) the single-spore isolate Kurokawa 21 produced in culture, (**C**) the single-spore isolate Kurokawa 22 produced in culture, and (**D**) naturally occurring sporulating lesions collected from the NIAES. Photos were taken one month after inoculation.

In a preliminary test conducted in 2005, two applications of fenarimol, sprayed 1 day before and 2 weeks after inoculation, controlled scab completely on trees inoculated with reference conidia from the NIAES. However, the control was 23.1%, 21.4%, and 21.1% on trees inoculated with conidia from the commercial orchards at Kurume, Ninaibaru, and Chikuzen in Fukuoka Pref., respectively. Decrease in fenarimol efficacy was clearly shown in inoculation tests carried out two times in the same year (Table 1 and Figure 1). High control of 94.6% was obtained against reference conidia from the NIAES. 100% control was recorded when conidia from Tagawa were inoculated. In contrast, values of the control were significantly lower and ranged from 2.1% to 77.7% when conidia from Ukiha 2, Kurokawa, Ukiha 1, Chikuzen, and Ninaibaru were inoculated.

In 2005 and 2006, 41 to 52 single-spore pure isolates were established from each of 7 orchards in Fukuoka mentioned above, and their sensitivity to fenarimol was tested in culture. The average EC_50_ values of this DMI varied from 0.147 mg/L for the isolates from Chikuzen to 1.012 mg/L for those from Kurokawa (Table 1). All of these values were higher than the baseline fenarimol sensitivity of 0.120 mg/L reported previously [24] and 0.142 mg/L retested in this study. In particular, the value 1.012 mg/L for the isolates from Kurokawa exhibited approximately 7 to 8 times higher than that for the baseline although the difference was not significant statistically. To demonstrate resistance more precisely, conidia of single-spore isolates produced on cellophane culture were employed as an inoculum source rather than those collected from naturally occurring sporulating lesions. The three isolates Kurokawa 9, Kurokawa 21 and Kurokawa 22 were chosen, because EC_50_ value of fenarimol for these isolates was 1.067 mg/L, 21.299 mg/L, and 5.906 mg/L, respectively (Table 2), higher than the concentration of 1 mg/L to distinguish less-sensitive isolates from sensitive ones [24]. Reference conidia from the NIAES were sensitive and completely controlled by fenarimol at each time point of disease assessment (Table 3). In contrast, control efficacy of fenarimol was inferior against the two isolates Kurokawa 21 (49.9%) and Kurokawa 22 (0.0%), 3 weeks after inoculation. The efficacy declined further against the three isolates including Kurokawa 9 (20.0%) under severe disease pressure 1 month after inoculation (Table 3). DMI resistance was thus confirmed in planta for the first time using single-spore isolates.

### 3.3. Inconsistency of Fenarimol Sensitivity Between in Planta and in Culture Tests

Fenarimol resistance was clearly demonstrated as above. Despite that, however, EC_50_ values for most of the other isolates from Kurokawa remained less than 1 mg/L, the discriminatory concentration. Moreover, no statistical difference was observed in average EC_50_ values for the isolates grouped based on sampling orchards (Table 1). It is important to note that the high EC_50_ values for some less-sensitive isolates influenced wide 95% CIs. The EC_50_ value of fenarimol was 0.328 ± 0.054 mg/L and 0.227 ± 0.047 mg/L, respectively, for isolates from Ukiha 1 and Ukiha 2, but control by fenarimol was only 21.6% and 2.1%, respectively, in inoculation tests (Table 1). Conversely, the EC_50_ value was 0.292 ± 0.056 mg/L and 0.253 ± 0.067 mg/L, respectively, for isolates from Ninaibaru and Tagawa. The conidia from these orchards were controlled 77.7% and 100%, respectively (Table 1). Thus, results from mycelial growth tests in culture were inconsistent with those from inoculation tests in planta.

In 2006, results from the inoculation tests using conidia sampled in the same year indicated decrease in fenarimol efficacy. The fungicide controlled 70.1% and 100%, against conidia from Kurokawa and the NIAES, respectively, whereas the control was only 25.0%, 31.7%, 32.4%, and 37.4%, respectively, against those from Usui, Kaho, Akizuki, and Akaike, Fukuoka Pref. (Table 1). In the trial, disease pressure was moderate to high because scab incidence ranged from 23.1% to 53.3% on DW-sprayed reference trees. For single-spore isolates from Kurokawa, reduced fenarimol sensitivity was obvious because the average EC_50_ value was 3.945 mg/L, exceeded 30 times higher than the baseline sensitivity in culture (Table 1). However, the value was 0.233 mg/L, 0.186 mg/L, 0.196 mg/L, and 0.278 mg/L, respectively, for the isolates from Usui, Kaho, Akizuki, and Akaike, which was less than the discriminative concentration of 1 mg/L (Table 1).

In 2017, 99 single-spore isolates, 19 to 20 for each, were established from five orchards in Imari, Saga Pref. and their sensitivity to fenarimol was examined in culture. The EC_50_ values were 2.675 mg/L and 1.125 mg/L, respectively, higher than the baseline for the two isolates, 1 Housui 18 and 1 Housui 21 (Table 2), but the values were lower than 1 mg/L for all of the other isolates. In the inoculation tests performed in 2018, however, sprays of fenarimol revealed 100% control against the isolate 1 Housui 18. Furthermore, no efficacy (−100% and −42.8% control) was recorded on the other two isolates, 1 Housui 13 and 2 Housui 11 (Table 2), respectively. Results from in planta tests did not coincide well with those from mycelial growth tests in culture. Therefore, the following three tests were carried out to interpret the inconsistency of both results.

### 3.4. Possible Source of the Inconsistency between in Planta and in Culture Tests

In 2007, infected leaves were collected from five trees grown in the middle and four corners in an orchard located in Ninaibaru, Fukuoka. Test (1) one single lesion was selected for each tree and conidial suspensions directly prepared from the lesion were used for droplet-inoculation onto leaves of potted trees sprayed with fenarimol preventatively. Control efficacy varied considerably (−100%, −60.1%, −14.1%, 0.0%, and 44.5%) suggesting irregular distribution of resistant isolates in the orchard tested. Test (2) soon after inoculation, 61 single-spore isolates were isolated from the same five lesions used as inoculum, and were tested for their fenarimol sensitivity in culture. Results showed that EC_50_ values were less than 1 mg/L, the discriminative concentration, for all of the isolates except only one isolate. Test (3) single-spore isolation was further conducted using the lesions newly produced by inoculation mentioned above and sensitivity of isolates was examined in culture again. Fifty-six and 71 isolates from fenarimol- and DW-treated leaves, five for each, respectively, were used for the tests. Very surprisingly, EC_50_ values of fenarimol were less than 1 mg/L for all of the 127 isolates irrespective of where they derived, i.e., either from fenarimol-treated leaves or from DW-treated leaves. It was strongly suggested that results from mycelial growth tests in culture are not very reliable for determining DMI sensitivity of this fungus.

### 3.5. Change of Cross-Resistance Pattern among DMI Fungicides with Time

In inoculation tests, reference conidia from the NIAES were controlled 100% by fenarimol, difenoconazole, and hexaconazole 3 weeks after inoculation (Table 4). Control efficacy of fenarimol on the conidia from Kurokawa was as low as 33.2% but both difenoconazole and hexaconazole completely controlled these conidia. However, 1 month after inoculation, sharp decrease occurred in the efficacy of fenarimol and hexaconazole but difenoconazole was still highly effective. Difenoconazole was superior to the other two DMIs at this stage in 2005 and incomplete cross-resistance among the three fungicides was thus found in planta.

Cross-resistance was further examined in culture using eight single-spore isolates including the three isolates (Kurokawa 9, Kurokawa 21, and Kurokawa 22) that showed resistance to fenarimol in planta as above (Table 3). The isolates Kurokawa 4, Kurokawa 9, Kurokawa 20, and Kurokawa 39 (Table 2) were sensitive to both difenoconazole and hexaconazole because EC_50_ values of these fungicides were within the range of baseline (Table 5). In contrast, the isolate Kurokawa 22 for which the EC_50_ of fenarimol was 5.906 mg/L, showed reduced sensitivity to difenoconazole and hexaconazole. Sensitivity of the isolates Kurokawa 18, Kurokawa 21, Kurokawa 22, and Kurokawa 26 was lower to hexaconazole.

In 2007, the conidia collected from five orchards in Imari, Saga Pref. were inoculated resulting in the control ranging from −22.7% to 36.7% for hexaconazole. In contrast, difenoconazole gave 71.4% to 100% control. These differences were significant based on comparison of the averages and their 95% CIs, 17.7 ± 21.04% (hexaconazole) and 88.4 ± 11.26% (difenoconazole), respectively, suggesting that difenoconazole was still effective at this stage in the fields although the efficacy of hexaconazole proved to decline largely. Next, single-spore isolates from these orchards in Imari were examined. Sensitivity to difenoconazole was not different significantly among the isolates but the average EC_50_ value of 0.260 mg/L was higher for isolates from 1 orchard than that for isolates from the other orchards. This result might have been an early sign of sensitivity shift for difenoconazole to resistance. In inoculation tests performed in 2008 using the conidia from Ninaibaru, Yasu, and Takagi, Fukuoka Pref., control efficacy of hexaconazole was 5.6, 7.9, and 91.5%, respectively, clearly indicating the lack of efficacy of this fungicide against the former two samples. On the contrary, difenoconazole revealed 84.0%, 90.9%, and 100% control, respectively, against these conidia. Hexaconazole and difenoconazole exhibited 77.4 and 100% control, respectively, against reference conidia from the NIAES. These results suggest that distribution of the hexaconazole-resistant isolates differed within the same prefectures.

In 2011, conidia collected from five leaves each of three commercial orchards in Yasu, Yasukawa, and Ninaibaru, Fukuoka and an experimental orchard in the Fruit Tree Research Institute, Uki, Kumamoto (FTRI) were droplet-inoculated on leaves of DMI- or DW-pretreated potted trees. Difenoconazole gave 100% and 97.8% control against the conidia from both Yasu and FTRI, respectively. In contrast, the control was 44.4% and 15.5%, significantly lower against the conidia from Yasukawa and Ninaibaru, respectively. Hexaconazole revealed no efficacy against the isolates from Yasu, Yasukawa, and Ninaibaru but exhibited 48.9% control against the isolates from the FTRI although this difference was not significant. Similar incomplete cross-resistance was recognized later when the single-spore isolate 2 Housui 1 was inoculated where difenoconazole revealed 100% control but fenarimol showed no efficacy (data not shown).

Most recently in 2018, 19 single-spores were isolated individually from lesions formed on leaves of inoculated pear trees after treatment with difenoconazole. The inoculum was sampled from 2 orchards in Yame, Fukuoka Pref. The EC_50_ of difenoconazole for 6 of 19 isolates ranged from 0.168 mg/L to 1.104 mg/L. These isolates were regarded as less-sensitive because the values were significantly higher than 0.001 mg/L for the reference isolate Yasato 2-1-1 [24] and the baseline 0.023 ± 0.142 mg/L as described above. Difenoconazole sensitivity of the other 13 isolates was within the range of baseline ranging from 0.001 mg/L to 0.154 mg/L). In inoculation tests conducted in 2018 and 2019, the two single-spore isolates 2 Housui 14 and 2 Housui 11 (Table 2), both isolated from Imari in 2017, were regarded as sensitive and resistant to difenoconazole, respectively, because control efficacy was 100% and −42.8% against these isolates, respectively.

### 3.6. Instability of Resistance

**Figure 2:**
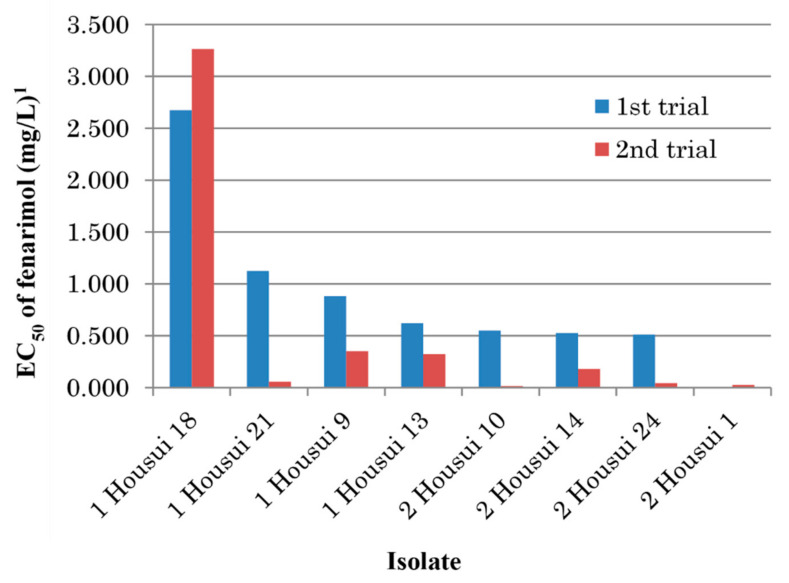
Change of fenarimol sensitivity in single-spore isolates of *Venturia nashicola* after storage at 4 °C in absence of the fungicide. ^1^ Average values of two replicates.

Mycelial growth tests were repeated on fenarimol-amended and unamended PDA using eight single-spore isolates collected from Kurokawa, Fukuoka, in 2005. As a result, except for the isolate Kurokawa 22, EC_50_ values of fenarimol decreased for all of the seven other isolates after storage in the absence of the fungicide (Table 6). Noticeably, the value for the isolate Kurokawa 21 decreased from 21.300 mg/L in the 1st trial to 1.721 mg/L in the 2nd trial, respectively. To confirm the instability of resistance in culture, similar experiments were conducted in 2018 using the eight other single-spore isolates obtained in the previous year. Sensitivity to fenarimol increased in seven isolates except the isolate 1 Housui 18 for which the EC_50_ value changed from 2.675 mg/L to 3.264 mg/L (Figure 2).

**Figure 3:**
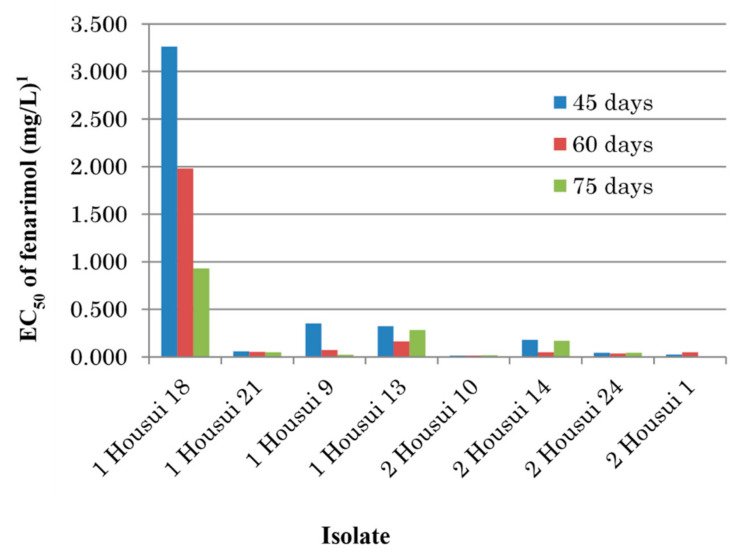
Influence of precultivation period against fenarimol sensitivity in single-spore isolates of *Venturia nashicola*. ^1^ Average values of two replicates.

To test the stability of decreased fenarimol sensitivity, eight representative isolates previously cultured on PDA at 20 °C in darkness for 45, 60, or 75 days, were tested for mycelial growth inhibition by transferring mycelial discs on fenarimol-amended PDA. The effect of prolonged cultivation period on the decrease in the EC_50_ values, i.e., recovery of the sensitivity was found in the isolate 1 Housui 18 (Figure 3).

### 3.7. Sequence Analysis of CYP51 Gene

Nucleotide sequences of the PCR amplicons from the 21 isolates used in this study (Table 2) showed 97–99% identity with GenBank AJ314649, sequence of the *CYP51* gene, encoding the DMI target sterol 14α-demethylase protein in the wild-type sensitive isolate JS-18 of *V. nashicola* [25]. Sequences of the deduced amino-acids of CYP51 were compared with GenBank CAC85409 reported by Cools et al. [25] and substitution of Y102N in addition to deletion of the Q359 were found in many isolates (Table 2), indicating two differences to GenBank sequence AJ314649 in all isolates not associated with a change in DMI sensitivity. Other substitutions found in less-sensitive or resistant isolates were G60S, Q110H, V131F, D291G, S310P, A320T, P324S, R366P, G428R, and G445D.

**Figure 4:**
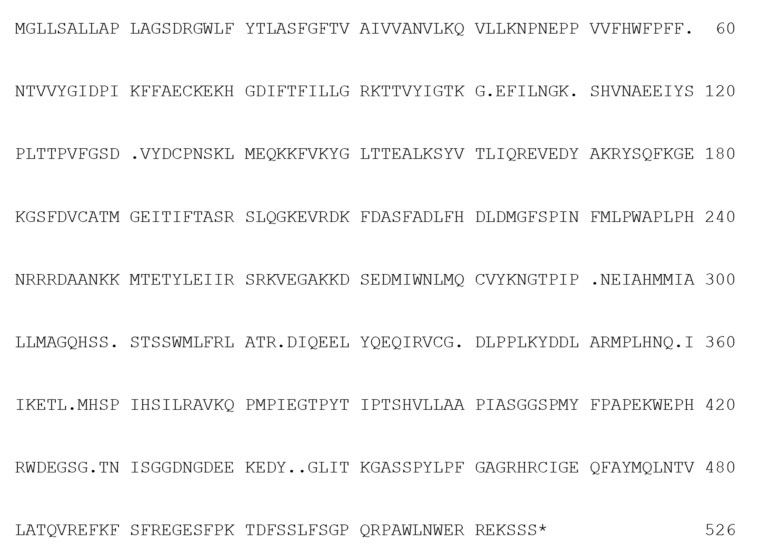
Translated deduced amino-acid sequence of *Venturia nashicola* CYP51 gene. Dots (.) indicate variants. Variants are summarized in Table 7. * Stop codon.

Subsequently, substitution of the Y446H was detected first from the two less difenoconazole-sensitive isolates, S Mizu 2 and S Mizu 4, collected from Yame, Fukuoka Pref. in 2018 (Table 2). Deletion of the Q359 and the G428R substitution were found in isolates irrespective of differential DMI sensitivity. Overall, genotypes of the *CYP51* gene varied largely leading to amino-acid substitutions (Figure 4 and Table 7 [31,32,33,34,35,36,37]) distinct from each other depending on origin, i.e., year and location of sampling of the isolates.

## 4. Discussion

The worldwide emergence of pathogens that are resistant to antifungal agents challenges human health and food security [38]. According to the Fungicide Resistance Action Committee (FRAC), DMI fungicides possess medium risk of resistance development (https://www.frac.info/, accessed on 23 June 2021) and failures of disease control against a variety of pathogens since the early 1980s have been reported. For scab control in Japanese pear, resistance has been managed mainly through restricting the number of DMI applications. Such an effort seemed to be effective as the field performance of DMIs has been maintained for about two decades. However, growers tended to spray DMIs more frequently than three times a year in some regions where disease pressure is generally high due to heavy precipitation. As a result, a decrease in fungicide efficacy became a concern and the development of resistance to fenarimol and hexaconazole was confirmed by inoculation tests. This was the first to confirm reduced DMI efficacy on scab by pathogen inoculation tests about 20 years after commencement of use in Japanese pear orchards. Resistance to hexaconazole and flusilazole was also reported in Korea where DMIs were sprayed more frequently [30].

Incomplete cross-resistance among DMIs is well known in the related fungus *V. inaequalis*, the causal agent of apple scab [39,40], and other fungi [41]. On the other hand, strong correlation of resistance to myclobutanil and tebuconazole has been found in this pathogen recently [42]. For Japanese pear scab, difenoconazole was intrinsically more active than other DMIs and the high efficacy of this fungicide was kept for years in fields where the others failed in control [22], but difenoconazole efficacy has also started to decline recently [43]. In Yame, Fukuoka Pref., where field performance of difenoconazole declined on scab, resistance development was also suspected in *Gymnosporangium asiaticum*, the Japanese pear rust fungus, through a retrospective cohort study [44]. Currently, the Fungicide Resistance Research Committee in the Phytopathological Society of Japan recommends limiting DMI applications within two to three times a year in a mixture with other effective fungicides carrying different mode of actions if resistant isolates have not been detected in the orchards (http://www.taiseikin.jp/, accessed on 23 June 2021). Cyprodinil (FRAC code 9, anilino-pyrimidines) and iminoctadine-albesilate (FRAC code M 07, multisite inhibitor) are generally used as a partner fungicide of DMIs (FRAC code 3) in a tank mixture. QoI (FRAC code 11, quinone-outside inhibitor) and SDHI (FRAC code 7, succinate dehydrogenase inhibitor) fungicides are also sprayed in addition to some other conventional fungicides.

Detection of resistance is critical to prevent control failure and design altered spray programmes as early as possible. When testing culturable fungi for their DMI sensitivity, mycelial growth tests on fungicide-amended culture medium are most commonly used. It was the case for *V. inaequalis* [45,46]. However, in this study on very slow-growing *V. nashicola*, reduced DMI sensitivity was not always reproduced in replicate experiments in culture, and sensitivity was often recovered after subculture, storage and/or when the cultivation period was prolonged in the tests. The instability of in vitro DMI resistance was previously reported in other fungi including *V. inaequalis* [47,48] and *Monilinia fructicola* [49,50]. For isolates of *V.* *inaequalis* cultured on DMI-amended PDA, reduced sensitivity to DMI-fungicides was maintained. In contrast, on fungicide-free media, the sensitivity of some, but not all, isolates increased [48], which implies DMI treatment induces expression of genes involved with resistance. It is a matter of interest whether such mechanisms may incur a fitness penalty which influences stability and could result in a decline of resistance in the field.

The association of a fitness penalty with DMI resistance has not been examined for *V. nashicola* isolates. For *V. inaequalis*, DMI resistance did not seem to be related to reduced fitness [51]. On the other hand, DMI-resistant populations of *V. inaequalis*, although still present, decreased in an apple orchard 3 years after the selection pressure was removed [52]. Myclobutanil sensitivity was recovered in populations of *V. inaequalis* after delayed-dormant copper treatment [53], and in Brazil, the frequency of resistant *M*. *fructicola* isolates decreased in populations after discontinuation of tebuconazole for 3 years [54].

In addition to slow growth of mycelia, conidial production of *V. nashicola* is extremely poor on culture medium, even worse than that of *V. inaequalis*. Moreover, the incubation period can be as long as 3 weeks to 1 month after inoculation under optimal environmental conditions. Despite that, two different tests, i.e., in vitro mycelial growth and in planta inoculation were performed in the present study. Importantly, we noticed an inconsistency between the results from these two tests indicating that comparison of the EC_50_ values of DMIs for pure isolates may not be very reliable for monitoring the sensitivity of field populations. When EC_50_ values above those of baseline sensitivity are measured, it might indicate the existence of resistant populations in the tested field. In contrast, even if values are within the range of baseline, it is possible that some less-sensitive individuals are included as a result of false negatives which might lead to the underestimation of resistance.

Inconsistency was often found when comparing results from tests in planta with those from tests in culture, due to the instability of resistance during storage or cultivation on media. Another factor that might explain this disparity is the irregular distribution of resistant isolates in pear orchards. Ishii et al. [9] found the isolates of *V. nashicola* resistant to MBC fungicides were detected irregularly from orchards. When several sampling methods were compared, remarkable differences were detected in the proportion of resistant isolates. This is closely related with biological context such as the lifestyle of this fungus in which conidia scatter mainly by rain splash and are not disseminated over long distances [55]. It was reported that the population subdivisions of *V. inaequalis* significantly differing in their sensitivity to myclobutanil were present in same apple orchards [56].

If conventional culture methods are not appropriate to monitor fungicide resistance, molecular-based methods might be useful as alternatives for a slow-growing and poor-sporulating fungus like *Venturia* in particular. The mechanism of MBC resistance was well characterized in *V. nashicola* [10,57] and various molecular methods such as PCR-RFLP (restriction fragment length polymorphism) analysis and ASPCR (allele-specific PCR) have been developed for the diagnosis of resistance [10]. However, mechanisms of the DMI resistance are not well known yet. For the related fungus *V. inaequalis*, the mechanism has been studied. Gerberich and Beckerman [58] found resistant isolates with larger PCR amplification bands consisting of the insertion or mutations of the *CYP51A1* gene and sensitive isolates with smaller amplification bands consisting of no insertions or mutations. Very recently, Yaegashi et al. [59] reported that Y137H substitution of the CYP51A was associated with low DMI sensitivity in Japanese isolates of *V. inaequalis*. In contrast, Schnabel and Jones [60] mentioned that overexpression of the target-site *CYP51A1* gene was an important mechanism of resistance, but other mechanisms of resistance also appeared to exist. Villani et al. [40] found the lack of cross-resistance between myclobutanil and difenoconazole and suggested that different mechanisms may govern resistance to different DMI fungicides in this pathogen. Cools and Fraaije [61] stated that these mechanisms can combine, and levels of resistance are often determined by combinations of CYP51 amino-acid alternations, *CYP51* gene overexpression and/or increased efflux. Furthermore, the combination of target-site mutations and over-expression causes a phenotype, with high levels of resistance [62].

Substitution at the equivalent amino-acid residue to Y136 and Y137 is the frequently reported CYP51 alteration in resistant isolates of both human and plant pathogenic fungi [61,63]. For *V. nashicola*, this substitution was not found but several mutations causing amino-acid substitutions or deletion such as G60S, Y102N, Q110H, V131F, D291G, S310P, A320T, P324S, Q359-, R366P, G428R, G445D, and Y446H were detected in the *CYP51* gene in some but not all resistant isolates in this study. Substitutions G445D and Y446H are at positions equivalent to alterations in DMI-resistant isolates of *Mycosphaerella fijiensis* and *Zymoseptoria tritici* (*Mycosphaerella graminicola*) [41,64,65], reported to be a key mutation for azole resistance [64,66]. Over 30 single nucleotide polymorphisms (SNPs) have been reported in CYP51, altering the protein structure and the binding activity of fungicides [61,67]. Accumulation of mutations generated various *CYP51* variants [61,68], and combination of mutations can result in alterations in the level of resistance to different DMI fungicides [63,67]. In the present study, the mutations leading to the substitutions such as Y102N found in less-sensitive isolates in the mid-2000s were not detected from isolates collected more recently in the late 2010s. Stepwise evolution of new resistant phenotypes in response to more active DMIs was reported in *M. graminearum* [41,67] but point mutations of the *CYP51* gene conferring DMI resistance are not very conserved in phytopathogenic fungi [39]. Therefore, it will be a future subject to examine whether these mutations are the cause of resistance and whether they can be utilized for resistance detection as a marker in *V. nashicola*.

It is most likely that other factors including increase of fungicide efflux from cells are involved in resistance. In our preliminary experiments, isolates which showed fenarimol resistance on fungicide-sprayed trees, were less-sensitive to the antibiotic cyclohexamide, an indicator of drug efflux pump activity but sensitive to chlorpromazine, a transporter modulator, in culture (Ishii et al. unpublished) indicating possible involvement of the ATP binding cassette (ABC) transporter family [68,69,70,71,72] in DMI resistance of this fungus. Low uptake of penconazole due to energy-dependent efflux was found to be the mechanism of resistance to this DMI in the laboratory mutant strains of *V. inaequalis* [73].

## 5. Conclusion and Future Prospects

In *V*. *nashicola*, results from mycelial growth tests using fungicide-amended culture medium are not very reliable for monitoring DMI resistance and molecular diagnosis of resistance seems to still be challenging in this fungus. Therefore, it is still necessary to confirm fungicide efficacy by inoculation tests on DMI-sprayed pear trees. The novel fungicide ipflufenoquin potentially possessing a new mode of action has been registered for pear scab recently in Japan. Additionally, the new DMI fungicide mefentrifluconazole [74], effective against scab with a reportedly more favorable toxicity profile [75], is now under development. Use of these fungicides in combination with disease-resistance inducers [76] and/or newly released scab-resistant pear cultivars [4,7] will be useful for integrated scab control in future.

## Figures and Tables

**Table 1:** Sensitivity of *Venturia nashicola* isolates to fenarimol in planta and in culture.

Year of Isolation	Location	Sensitivity to Fenarimol in Planta (Control, %) ^1^	Sensitivity to Fenarimol in Culture (Average EC_50_, mg/L) ^2^
2005	Ukiha 2, Fukuoka	2.1 *	0.227
	Kurokawa, Fukuoka	3.7 *	1.012
	Ukiha 1, Fukuoka	21.6 *	0.328
	Chikuzen, Fukuoka	37.8 *	0.147
	Kurume, Fukuoka	55.3	0.370
	Ninaibaru, Fukuoka	77.7 *	0.292
	Tagawa, Fukuoka	100	0.253
	NIAES, Ibaraki ^3^	94.6	0.142
2006	Usui, Fukuoka	25.0	0.233
	Kaho, Fukuoka	31.7	0.186
	Akizuki, Fukuoka	32.4	0.196
	Akaike, Fukuoka	37.4	0.278
	Kurokawa, Fukuoka	70.1	3.945
	NIAES, Ibaraki ^3^	100	0.142

^1^ Conidial suspensions prepared from naturally occurring sporulating lesions were inoculated on the leaves of fenarimol (30 mg AI/L)-treated and untreated pear trees. Control (%) was calculated based on the scab incidence. ^2^ Forty-one to 52 single-spore isolates from each location were used for the mycelial growth tests. EC_50_ means 50% effective concentration to inhibit growth. ^3^ Sensitive reference. * Significantly different from the value of control (%) against the reference National Institute for Agro-Environmental Sciences (NIAES) isolates based on comparison of the average and 95% confidence intervals (CIs).

**Table 2:** Collection information, DMI sensitivity, and deduced amino-acid change of CYP51 for the representative single-spore isolates of *Venturia nashicola* used in this study.

Isolate	Year of Isolation	Location	Response to Fenarimol or Difenoconazole in Culture ^1^	Sensitivity to Fenarimol or Difenoconazole in Culture (EC_50,_ mg/L) ^2^	Deduced Amino-Acid Change of CYP51
Kurokawa 4	2005	Asakura, Fukuoka	Sensitive	0.151	Q359 (-), G428R
Kurokawa 39	2005	Asakura, Fukuoka	Sensitive	0.166	Q359 (-), G428R
Kurokawa 18	2005	Asakura, Fukuoka	Less-sensitive	1.948	Y102N, D291G, Q359 (-), G445D
Kurokawa 20	2005	Asakura, Fukuoka	Less-sensitive	0.504	Y102N, A340T, Q359 (-), R366P
Kurokawa 26	2005	Asakura, Fukuoka	Less-sensitive	1.970	Y102N, Q110H, V131F
Kurokawa 9	2005	Asakura, Fukuoka	Resistant ^3^	1.067	Q359 (-), G428R
Kurokawa 21	2005	Asakura, Fukuoka	Resistant	21.299	G60S, Y102N, Q359 (-), G428R
Kurokawa 22	2005	Asakura, Fukuoka	Resistant	5.906	Y102N, S310P, P324S, Q359 (-), G428R
2 Housui 1	2017	Imari, Saga	Sensitive	0.003	Q359 (-)
1 Housui 9	2017	Imari, Saga	Less-sensitive	0.884	Q359 (-)
1 Housui 18	2017	Imari, Saga	Less-sensitive	2.675	Q359 (-)
1 Housui 21	2017	Imari, Saga	Less-sensitive	1.125	Q359 (-)
2 Housui 10	2017	Imari, Saga	Less-sensitive	0.551	Not tested
2 Housui 14	2017	Imari, Saga	Less-sensitive	0.526	Q359 (-)
2 Housui 24	2017	Imari, Saga	Less-sensitive	0.512	Q359 (-)
1 Housui 13	2017	Imari, Saga	Resistant	0.619	Q359 (-)
2 Housui 11	2017	Imari, Saga	Resistant	0.033	Not tested
H Mizu 3	2018	Yame, Fukuoka	(Sensitive)	(0.000)	Q359 (-)
H Mizu 5	2018	Yame, Fukuoka	(Sensitive)	(0.044)	Q359 (-)
S Mizu 2	2018	Yame, Fukuoka	(Less-sensitive)	(1.104)	Q359 (-), Y446H
S Mizu 3	2018	Yame, Fukuoka	(Less-sensitive)	(0.196)	Q359 (-)
S Mizu 4	2018	Yame, Fukuoka	(Less-sensitive)	(0.257)	Q359 (-), Y446H
S Mizu 5	2018	Yame, Fukuoka	(Less-sensitive)	(0.593)	Q359 (-)

^1^ Response to difenoconazole is shown in parenthesis. ^2^ EC_50_ value of difenoconazole is shown in parenthesis. ^3^ Determined based on reduction in fungicide efficacy in planta.

**Table 3:** Control efficacy of fenarimol (30 mg AI/L) on single-spore isolates of *Venturia nashicola* in planta.

Isolate	Time After Inoculation	Control (%)	Scab Incidence (%) on DW-Sprayed Reference Trees
Kurokawa 9	3 weeks	85.7	93.3
	1 month	20.0	100.0
Kurokawa 21	3 weeks	49.9	93.3
	1 month	6.7	100.0
Kurokawa 22	3 weeks	0.0	86.7
	1 month	0.0	100.0
NIAES ^1^	3 weeks	100.0	33.3
	1 month	100.0	73.3

^1^ Conidia directly collected from naturally occurring sporulating lesions.

**Table 4:** Control efficacy of the three DMI fungicides against *Venturia nashicola* conidia in planta.

Timing of Disease Assessment			Origin of Conidia Inoculated	Treatment	Scab Incidence (%)	
3 weeks after inoculation			Kurokawa	Fenarimol ^1^	33.2	19.5
				Difenoconazole ^2^	100	0.0
				Hexaconazole ^3^	100	0.0
				Distilled water		29.2
			NIAES	Fenarimol	100	0.0
				Difenoconazole	100	0.0
				Hexaconazole	100	0.0
				Distilled water		17.9
1 month after inoculation			Kurokawa	Fenarimol	−14.7	88.4
				Difenoconazole	100	0.0
				Hexaconazole	21.7	60.4
				Distilled water		77.1
			NIAES	Fenarimol	65.9	20.9
				Difenoconazole	100	0
				Hexaconazole	90.9	5.6
				Distilled water		61.3

^1^ 30 mg AI/L. ^2^ 25 mg AI/L. ^3^ 10 mg AI/L.

**Table 5:** Sensitivity of *Venturia nashicola* isolates to the three DMI fungicides in culture.

Isolate	EC_50_ (mg/L) of Fungicide		
	Fenarimol	Difenoconazole	Hexaconazole
Kurokawa 4	0.151	<0.010	0.020
Kurokawa 9	1.067	<0.010	<0.010
Kurokawa 18	1.948	<0.010	0.072
Kurokawa 20	0.504	<0.010	0.023
Kurokawa 21	21.299	0.032	0.113
Kurokawa 22	5.906	3.199	0.091
Kurokawa 26	1.970	<0.010	0.028
Kurokawa 39	0.166	<0.010	0.022
Baseline	0.142 ± 0.215	0.023 ± 0.142	0.007 ± 0.016

**Table 6:** Change of fenarimol sensitivity in single-spore isolates of *Venturia nashicola* after storage at 4 °C in absence of the fungicide.

Isolate	Sensitivity to Fenarimol	EC_50_ (mg/L) of Fenarimol	
		1st trial	2nd trial
Kurokawa 4	Sensitive	0.151	0.028
Kurokawa 39	Sensitive	0.166	0.131
Kurokawa 18	Less-Sensitive	1.948	0.973
Kurokawa 20	Less-Sensitive	0.504	0.347
Kurokawa 26	Less-Sensitive	1.970	0.680
Kurokawa 9	Resistant ^1^	1.067	0.263
Kurokawa 21	Resistant ^1^	21.299	1.721
Kurokawa 22	Resistant ^1^	5.906	7.879

^1^ Reduction in fenarimol efficacy was also confirmed in planta (Table 3).

**Table 7:** Amino-acid (AA) positions in *CYP51* where mutations in the isolates of *Venturia nashicola* were sequenced. For each AA position the corresponding mutation and its eventual orthology are shown. SEPTTR: *Zymoseptoria tritici*; ERYSGT: *Blumeria graminis* f. sp. *tritici*; BRYSGH: *Blumeria graminis* f. sp. *hordei*; UNCINE: *Erysiphe necator*; MONIFG: *Monilinia fructigena*; LEPTNO: *Parastagonospora nodorum*; CERBE: *Cercospora beticola*. Table references: ^1^ Stammler et al. [31]; ^2^ Wyand and Brown [32]; ^3^ Rallos and Baudoin [33]; ^4^ Chen et al. [34]; ^5^ Pereira et al. [35]; ^6^ Cools et al. [36]; ^7^ Spanner et al. [37].

AA Position	Mutation	Orthology
60	G60S	
102	Y102N	
110	Q110H	
131	V131F	V131F is placed between D130 (SEPTTR_D134 ^1^) and Y133 (ERYSGT ^2^/BRYSGH ^2^/UNCINE ^3^/MONIFG ^4^/_Y136 or LEPTNO_Y144 ^5^)
291	D291G	
310	S310P	
324	P324S	
340	A340T	
359	Q359-	
366	R366P	
428	G428R	
445	G445D	SEPTTR_G460 ^6^
446	Y446H	CERBE_Y464 ^7^

## Data Availability

Not applicable.
